# A rare case of facial cutaneous Rosai-Dorfman disease clinically mimicking basal cell carcinoma followed by multiple myeloma after 2 years

**DOI:** 10.25122/jml-2023-0337

**Published:** 2024-02

**Authors:** Baraa Amir, Amaar Amir, Salwa Sheikh

**Affiliations:** 1 Imam Abdulrahman Bin Faisal University, Dammam, Saudi Arabia; 2 Pathology Services, Johns Hopkins Aramco Healthcare, Dhahran, Saudi Arabia

**Keywords:** Rosai-Dorfman disease, carcinoma, multiple myeloma, sinus histiocytosis with massive lymphadenopathy, emperipolesis, RDD, Rosai-Dorfman disease, BMI, Body Mass Index, SCT, Stem Cell Therapy, RVD, Lenalidomide, Bortezomib, Dexamethasone, CRDD, Cutaneous Rosai-Dorfman disease, FISH, Fluorescence in situ hybridization

## Abstract

Rosai-Dorfman disease (RDD) is a rare non-Langerhans cell histiocytosis disorder characterized by the proliferation of histiocytes within the lymph nodes. Extranodal involvement can occur; however, only 10% of extranodal RDD involve the skin. We present a unique case of a 66-year-old woman with cutaneous RDD followed by the development of multiple myeloma (MM). To our knowledge, this is only the second reported case where RDD preceded a diagnosis of MM, with the first documented instance occurring in 2018. The patient presented to the dermatology clinic with a 5-year history of painless, solitary lesion over the right cheek. Local examination revealed a single 6 mm x 7 mm well-circumscribed pearly telangiectatic lesion resembling basal cell carcinoma over the right nasolabial fold and cheek. The lesion was excised with a 3 mm circumferential margin. Histopathology showed a mixed lymphohistiocytic cell infiltrate with emperipolesis and immunohistochemical staining patterns consistent with RDD. Two years later, the patient presented with hip pain and was diagnosed with MM. She was treated with lenalidomide, bortezomib, and dexamethasone, and was later maintained on lenalidomide. Our case adds to the limited evidence suggesting a potential association between RDD and MM. Further research in this field is required to promptly identify and manage patients with such a presentation in the future.

## INTRODUCTION

Rosai-Dorfman disease (RDD) was originally described in 1965, involving four cases presenting with lymphadenopathy and sinus histiocytosis in children and young adults. It is a rare non-Langerhans cell histiocytosis characterized by the proliferation of histiocytes within lymph nodes. Although extranodal involvement occurs in approximately 43% of cases, only 10% include cutaneous manifestations, and merely 3% are exclusively localized on the skin [1].

RDD has been associated with various conditions, including viruses, immune disorders, and neoplasias. However, its occurrence with multiple myeloma (MM) is exceptionally rare, with only one concurrent case reported in 2018. Herein, we report a rare case of a 66-year-old woman presenting with facial cutaneous RDD clinically mimicking basal cell carcinoma who developed MM 2 years later.

## CASE PRESENTATION

A 66-year-old Saudi woman presented to our dermatology clinic with a painless, solitary lesion on her right cheek. The lesion had been present for 5 years and had gradually grown in size. The patient was asymptomatic and did not have a history of fever, chills, night sweats, significant weight loss, or any other associated symptoms. The patient was postmenopausal, never smoked, and her past medical history was positive for type 2 diabetes mellitus, obesity (body mass index [BMI]: 36.7 kg/m^2^), beta thalassemia trait, arthritis, and chronic back pain. The surgical history was positive for a hysterectomy. Examination revealed a single, well-defined, pearly, telangiectatic lesion measuring 6mm x 7mm on the right nasolabial fold extending to the cheek. There were no other lesions on her face. Based on the clinical presentation, the initial impression was that of basal cell carcinoma (BCC). The patient's laboratory investigations were all within normal limits. She was referred to plastic surgery, where she underwent excision.

**Figure 1:**
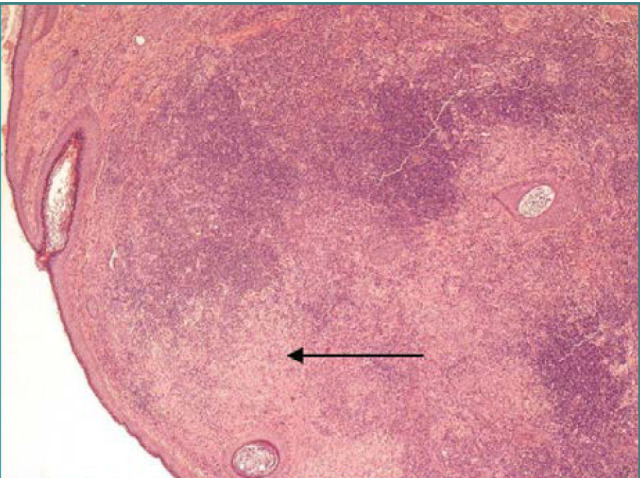
Histopathological examination of the skin with dense lymphoplasmacytic infiltration and epithelioid histiocyte aggregates (Hematoxylin-eosin stain, original magnification 4x.

**Figure 2:**
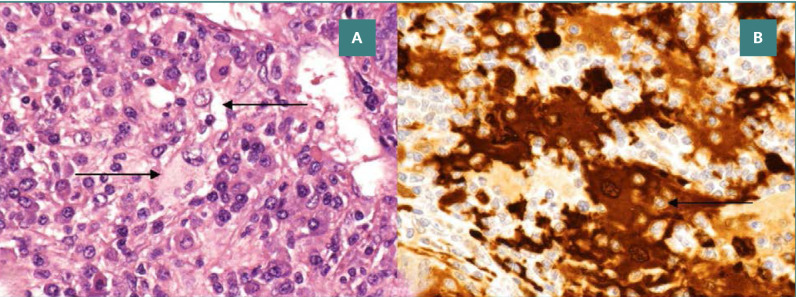
A, Large histiocytes with abundant pale to eosinophilic cytoplasm showing engulfed intact lymphocytes (emperipolesis) (Hematoxylin-eosin stain, 40x magnification); B, S-100 protein staining highlighting epithelioid histiocytes with distinctive cytoplasmic staining and clear halos around engulfed lymphocytes (S-100 stain, 40x magnification).

Histopathological examination demonstrated a mixed cell population composed predominantly of mature lymphocytes with intermixed plasma cells involving the dermis. Additionally, there were large epithelioid histiocytes with enlarged, round to oval nuclei and abundant eosinophilic cytoplasm, often containing engulfed intact inflammatory cells, a process known as emperipolesis (Figure 1, 2). These cells were positive for S100, CD68, and CD30 by immunohistochemical staining. The cells were negative for Melan-A, CD1a, cytokeratin, CD15, MUM1, and PD1 antigens. The Kappa and Lambda in situ stains demonstrated polyclonal plasma cell population. The mixed lymphohistiocytic cell infiltrate, emperipolesis, and immunohistochemical staining patterns were consistent with Rosai-Dorfman disease.

The patient remained well for 2 years until she developed hip pain. Laboratory investigations and peripheral blood smear revealed microcytic hypochromic anemia with anisopoikilocytosis and rouleaux formation. Platelet and white blood cell counts were normal. A pelvic x-ray revealed numerous lytic lesions. An M-spike was detected on protein electrophoresis; there was elevated serum IgG at 5290 mg/dL and elevated urine protein at 364 mg in a 24-hour collection period. Quantification of free light chains yielded elevated kappa-free light chains at 39.8 mg/l, a normal lambda-free light chain at 1.05 mg/l, and an increased kappa-to-lambda ratio of 37.9. Bone marrow biopsy from the patient’s iliac crest showed approximately 40% plasma cells highlighted by CD138 immunohistochemical stain. These plasma cells displayed an aberrant phenotype with kappa light chain restriction, as confirmed by flow cytometry and in situ hybridization. DNA analysis revealed a normal diploid population. Further analysis using fluorescence in situ hybridization (FISH) identified a plasma cell clone with trisomies 9, 11, and 15, which likely indicates a hyperdiploid clone. Additionally, monosomy 13 was detected. The combined findings were consistent with multiple/plasma cell myeloma.

The patient was offered stem cell therapy (SCT); however, she declined this treatment modality. She was treated with lenalidomide, bortezomib, and dexamethasone (RVD) therapy and went into remission, followed by a maintenance regimen of lenalidomide.

## DISCUSSION

Rosai-Dorfman disease (RDD), first described in 1965, is a rare non-Langerhans cell histiocytosis. Initially recognized as lymphadenopathy with sinus histiocytosis in children and young adults, it was later defined by Rosai and Dorfman in 1969 as a distinct disease termed “sinus histiocytosis with massive lymphadenopathy” [2,3]. It is a rare non-Langerhans cell histiocytosis characterized by histiocytic proliferation within the lymph nodes. Extra-nodal sites are involved in approximately 40% of cases, with skin being the most common extra-nodal site of involvement, representing only 10% of RDD cases. Clinical features vary depending on the location of the disease. Classic/nodal RDD and cutaneous RDD (CRDD) often have a benign course with minimal symptoms. Conversely, disseminated RDD can be more aggressive and involve multiple organ systems in 20% of cases. The revised classification of histiocytic disorders categorizes RDD as either the ‘R’ group (nodal and extra-nodal disease) or the ‘C’ group (cutaneous disease) based on the location [4]. The typical RDD clinical presentation is bilateral painless lymphadenopathy with associated fever, high erythrocyte sedimentation rate, hypergammaglobulinemia, and immunologic dysfunction [5]. The etiology remains unknown and idiopathic. However, associations have been reported with viral infections, immune disorders, and some cancers, particularly lymphomas. Additionally, RDD has been observed after bone marrow transplants for leukemia and myelodysplastic syndrome. There are also documented cases of RDD diagnosed concurrently with clear cell sarcoma, malignant histiocytosis, and IgG4 disease [3,6,7]. To our knowledge, only one case report (2018) describes concurrent RDD and multiple myeloma, making our case the second documented instance of this rare association [8].

Histopathological examination confirmed the diagnosis of Rosai-Dorfman disease. The classic finding is an accumulation of histiocytes with large round to oval nuclei and moderate to abundant eosinophilic cytoplasm. These cells are often seen engulfing intact inflammatory cells referred to as emperipolesis [7]. Approximately one-third of these cases show clonal mitogen-activated protein kinase/extracellular signal-regulated kinase (MAPK/ERK pathologic alterations). This alteration suggests a pathogenesis that is similar to Langerhans cell histiocytosis and Erdheim-Chester disease [9]. In Erdheim-Chester disease, the *BRAF* V600E mutation is frequently observed; however, testing for this specific mutation revealed a wild-type result. Some recent studies also identified the presence of *NRAS, KRAS, ARAF*, and *MAP2K1* mutations; and familial cases may show germline mutations in the *SLC29A3* gene [3].

RDD generally has a favorable prognosis, particularly for cases with nodal and cutaneous involvement, with only a subset of patients, mostly with multifocal disease, showing aggressive clinical course. The International Registry for Rare Histiocytic Disorders is an ongoing effort to improve understanding of the clinical features, treatment options, and outcomes for RDD and other rare histiocytic disorders. To date, only one prior case report of simultaneous cutaneous RDD and MM was published in 2018. Our case adds significantly to the limited literature, presenting the first reported instance of cutaneous RDD, followed by the subsequent development of MM two years later. Given the limited data on this rare co-occurrence, further research is warranted. Comprehensive genetic analysis could be instrumental in determining whether RDD and MM share a valid molecular link, or if their coexistence is simply incidental.
